# Microarray analysis of expression of cell death-associated genes in rat spinal cord cells exposed to cyclic tensile stresses in vitro

**DOI:** 10.1186/1471-2202-11-84

**Published:** 2010-07-22

**Authors:** Kenzo Uchida, Hideaki Nakajima, Takayuki Hirai, Takafumi Yayama, Ke-Bing Chen, Shigeru Kobayashi, Sally Roberts, William E Johnson, Hisatoshi Baba

**Affiliations:** 1 Department of Orthopaedics and Rehabilitation Medicine, Fukui University Faculty of Medical Sciences, Shimoaizuki 23, Matsuoka, Fukui 910-1193, Japan; 2 Institute for Science & Technology in Medicine, Keele University at the RJAH Orthopaedic Hospital, Oswestry, Shropshire SY10 7AG, UK

## Abstract

**Background:**

The application of mechanical insults to the spinal cord results in profound cellular and molecular changes, including the induction of neuronal cell death and altered gene expression profiles. Previous studies have described alterations in gene expression following spinal cord injury, but the specificity of this response to mechanical stimuli is difficult to investigate in vivo. Therefore, we have investigated the effect of cyclic tensile stresses on cultured spinal cord cells from E15 Sprague-Dawley rats, using the FX3000^® ^Flexercell Strain Unit. We examined cell morphology and viability over a 72 hour time course. Microarray analysis of gene expression was performed using the Affymetrix GeneChip System^®^, where categorization of identified genes was performed using the Gene Ontology (GO) and Kyoto Encyclopedia of Genes and Genomes (KEGG) systems. Changes in expression of 12 genes were validated with quantitative real-time reverse transcription polymerase chain reaction (RT-PCR).

**Results:**

The application of cyclic tensile stress reduced the viability of cultured spinal cord cells significantly in a dose- and time-dependent manner. Increasing either the strain or the strain rate independently was associated with significant decreases in spinal cord cell survival. There was no clear evidence of additive effects of strain level with strain rate. GO analysis identified 44 candidate genes which were significantly related to "apoptosis" and 17 genes related to "response to stimulus". KEGG analysis identified changes in the expression levels of 12 genes of the mitogen-activated protein kinase (MAPK) signaling pathway, which were confirmed to be upregulated by RT-PCR analysis.

**Conclusions:**

We have demonstrated that spinal cord cells undergo cell death in response to cyclic tensile stresses, which were dose- and time-dependent. In addition, we have identified the up regulation of various genes, in particular of the MAPK pathway, which may be involved in this cellular response. These data may prove useful, as the accurate knowledge of neuronal gene expression in response to cyclic tensile stress will help in the development of molecular-based therapies for spinal cord injury.

## Background

Mechanical stresses applied to the spinal cord can potentially induce profound and irreversible paresis, secondary to induced pathological changes such as dysfunction and loss of neurons, impairment of neuronal cell survival mechanisms and protein synthesis, neuronal cell necrosis and apoptosis [1,2]. Examples of such mechanically induced spinal cord damage include not only spinal cord compression but distraction insult [3,4]; however, it is likely that tensile stresses form an important part of many injuries of the spinal cord. The primary mechanical event, which may occur in less than a second, can initiate a cascade of molecular and cellular events such as changes in gene expression, which may then influence cell function over minutes to hours or a much longer period. For example, transient disruption of Ca^2+ ^homeostasis may be an early event in a series of aberrant signaling cascades that ultimately lead to cellular dysfunction or cell death. Extended consequences of this molecular cascade include changes in gene expression levels that are necessary for cell recovery or cell death [5-8].

Changes in the expression of several immediate early response genes have been documented in various in vivo models of spinal cord injury using microarray analysis [9-11]. These included the up regulation of transcription factors, suggesting that the expression of many other genes is potentially regulated after traumatic insult. Indeed, the differential and post-traumatic expression levels of several genes have been explored in vivo in an attempt to stabilize, both biologically and functionally, the spinal cord once injured [12,13]. However, these in vivo experimental settings for studying the response of neuronal systems to mechanical injury suffer from several disadvantages over in vitro experimentation. For example, mechanical stress can cause additional or unexpected tissue or cell reactions such as activation of resident inflammatory cells or invasion of foreign cells from the periphery [14]. The complexity of the in vivo situation may also result in a limited accessibility to specific areas of tissue or cell type of interest, preventing real-time and spatial measurement of biological or mechanical parameters [15]. Thus, in vitro models of the spinal cord stimuli can be useful to gain a better understanding of the specific neuronal response to mechanical stress.

One approach to determine the pathophysiology of mechanical-stress-related spinal cord damage is to investigate the in vitro response of neuronal cells to loading. The use of neuronal cell culture models allows for better control of the extracellular environment, is relative easy to manipulate, and permits for repeated access to neural cells for specific analysis. The spinal cord and neurons are always subjected to mechanical stress including tensile stresses, during spine movement. Longitudinal vertebral distraction and the physiological tension zone [16] of the spinal cord are closely correlated each other when the spine is subjected to flexural positioning [17,18] and excessive kyphosis in the thoracic vertebrae [19]. The Flexercell Strain Unit (FX3000^®^, Flexercell International, Hillsborough, NC) is a cell-stretching apparatus that allows application of cyclic tensile force to cultured cells. The system has been used to elucidate the mechanism of mechanical signaling in various types of cells [20-22]. In our previous study using this equipment [23], we investigated the in vitro effects of cyclic tensile stress on cultured spinal cord cells, with a special focus on the expressions of neurotrophins and their receptor genes. The results of that study showed that the application of tensile stress increased the expression levels of nerve growth factor, brain-derived neurotrophic factor, trkB, p75 neurotrophin receptor (p75NTR), glial cell line-derived neurotrophic factor, and caspase-9 mRNAs in the acute phase, followed by increased lactate dehydogenase release and induction of necrotic cell death. These findings led us to investigate further the expression of several genes related to cell death in cultured spinal cord cells under cyclic tensile stress.

The present study was thus designed to examine further the molecular changes and gene expression profiles in cultured spinal cord cells using the above cell-stretching apparatus and DNA microarray technology in order to provide a more complete picture of the changes in the expression of specific genes involved in neuronal response to cyclic tensile stress.

## Results

### Cyclic tensile stress induced spinal cord cell death

**Figure 1:**
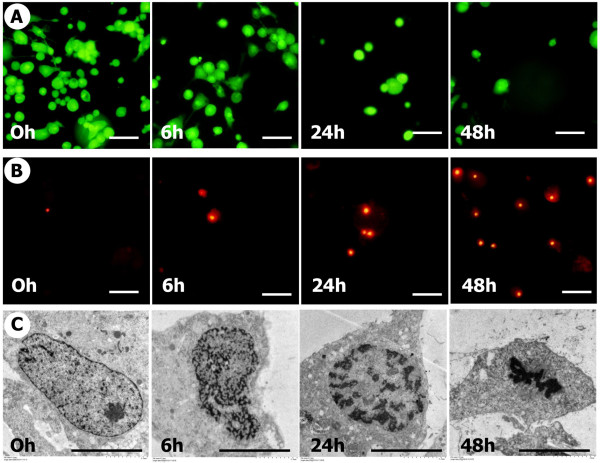
**The application of cyclic tensile stress induced apoptotic cell death in spinal cord cells**. Representative serial photomicrographs are shown of cells exposed to a tensile stress of 10% strain at 0.5 Hz at 0, 6, 24, and 48 hours (A, B). The number of green-stained living cells decreased (A: top row) while the number of red-strained dead cells increased (B: middle row) in a time-dependent manner during cyclic tensile stress application. Transmission electron microscopy (TEM) examination (C: bottom row) showed that all cells at 0 hour appeared viable, with large nuclei, and dotted with chromatin and abundant rough endoplasmic reticulum, while some cells at 6 hours showed deformity of nuclei and cytoplasm. TEM at 24 hours showed some cells with condensed and fragmented nuclei and condensed chromatin, and the change progressed at 48 hours (C: bottom row). Bar = 100 μm (A, B), 50 μm (C).

Under the condition of 10% tensile strain (typically found in spinal cord injuries [19]) at a frequency of 0.5 Hz (Figure 1A, B), the proportion of living green-stained spinal cord cells decreased in a time-dependent manner, whereas that of dead red-stained cells increased simultaneously. Transmission Electron Microscopy (TEM) examination showed that all cells at 0 hours appeared viable, with large nuclei, and dotted with chromatin and an abundant rough endoplasmic reticulum. In contrast, in cultures subjected to a tensile stress of 10% strain at 0.5 Hz some cells appeared to show deformity of the nuclei and cytoplasm at 6 hours, and chromatin condensation and fragmentation were observed at 24 hours. These morphological changes were indicative of the start of apoptosis and progressed at 48 hours (Figure 1C).

**Figure 2:**
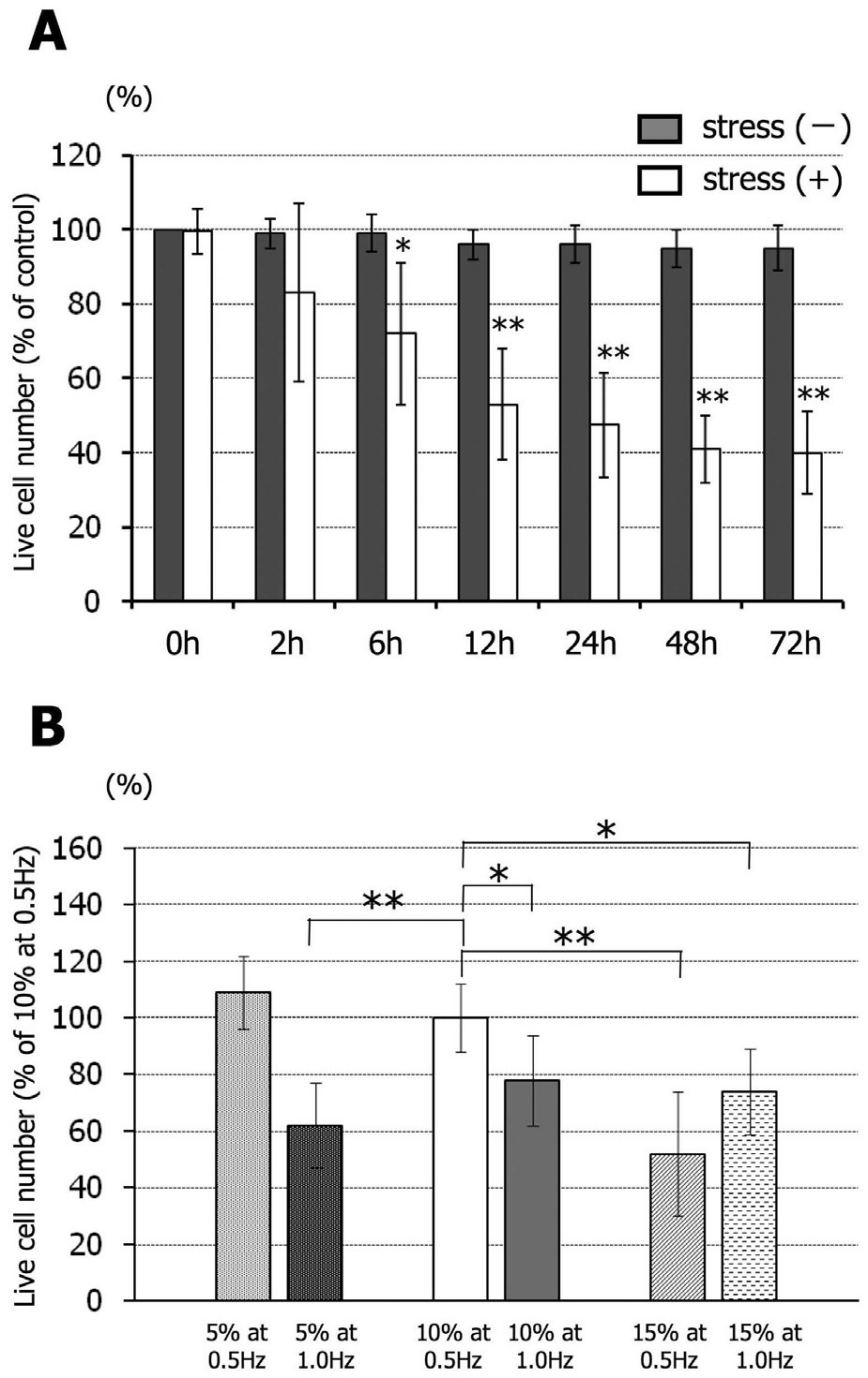
**The survival rate of spinal cord cells was dependent on the level, frequency and duration of the applied tensile strain**. The survival rate (%) of living cells during the application of a standardized cyclic tensile stress (10%, 0.5 Hz) compared with stress-free baseline (A). Gray bar: stress-free condition; white bar: cyclic tensile stress condition. The survival rate (%) of living cells after 6 hours at three different strain levels of 5%, 10%, and 15% and two different strain frequencies of 0.5 Hz and 1 Hz compared with that at the standardized strain level of 10% at 0.5 Hz frequency (B). Data are expressed as mean ± SEM of 6 experiments. *P < 0.05, **P < 0.01.

The cell survival rate of cultures under this cyclic strain as a proportion of that of non strained cultures decreased from 83 ± 24% at 2 hours to 72 ± 19% at 6 hours, 53 ± 15% at 12 hours, 48 ± 14% at 24 hours, 41 ± 9% at 48 hours, and 40 ± 11% at 72 hours (Figure 2A). This decrease in cell survival became significant after 6 hours. Figure 2B shows the cell survival rates as a % of that seen in cultures subjected to a designated standard tensile stress, i.e. 10% strain and 0.5 Hz, after 6 hours at the three different strain levels of 5%, 10%, and 15% and the two different strain rates of 0.5 Hz and 1 Hz. The cell survival rate in cultures subjected to a 10% strain but at a frequency of 1 Hz was significantly lower than that seen at this standard level. Therefore, increasing the strain rate independently of the strain level was associated with increased spinal cord cell death. Conversely, the cell survival rate in cultures subjected to a 15% strain level at a frequency of 0.5 Hz was also significantly lower than that seen at the standard level. Therefore, increasing the strain level independently of the strain rate was also associated with increased spinal cord cell death. There was no clear evidence of additive effects of strain level with strain rate.

### Cluster analysis of gene expression profiles

**Figure 3:**
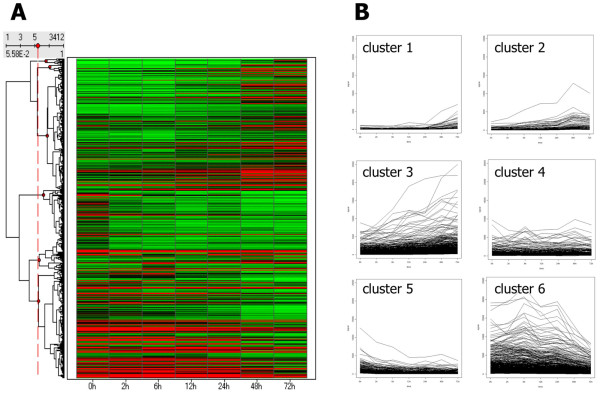
**Gene expression profiles in spinal cord cells after the application of cyclic tensile stress**. The hierarchial clustering data are presented graphically as a heatmap (A). These genes were subsequently grouped into six subclusters (cluster 1-6) according to the time course of gene expression pattern (B).

There was altered expression of 3,412 genes after the application of a cyclic tensile stress of 10% at 0.5 Hz. These 3, 412 genes were profiled using hierarchical cluster analysis, based on similarities among their expression patterns in a time course manner. Accordingly, they were divided into 6 clusters (Figure 3). Cluster 1 comprised 67 genes, cluster 2 comprised 102 genes, cluster 3 comprised 1,240 genes, cluster 4 comprised 355 genes, cluster 5 comprised 499 genes, and cluster 6 comprised 1,149 genes. As shown, genes of clusters 1, 2 and 3 were upregulated in a time-dependent manner under cyclic tensile stress, whereas those of cluster 4 were upregulated in a time-independent manner. Genes of clusters 5 and 6 were downregulated in a time-dependent manner under cyclic tensile stress during the 72 hours.

### Identification of upregulated genes through Gene Ontology (GO) analysis

Based on the results of clustering analysis, we tested the genes by all GO terms within the biological process in each of the clusters 1-6, and subsequently identified the upregulated genes related to cyclic tensile stress among clusters 1, 2 and 3. The gene related to "apoptosis" belongs to cluster 3 significantly among the cluster 1-3 and the gene related to "response to stimulus" belonged to cluster 1 significantly among the cluster 1-3. Candidate genes related significantly to "apoptosis" among the terms of a biological process were 44 genes in cluster 3, and those significantly related to "response to stimulus" were 17 genes in cluster 1. These genes are listed in Tables 1 and 2, including the relative signal intensity, which was expressed relative to that at 0 hours (boldface show more than 2-fold). Forty-four genes related to "apoptosis" were expressed in the early phase (cluster 3), whereas 17 genes related to "response to stimulus" were expressed in the late phase (cluster 1).

**Table 1:** List of "apoptosis" genes selectively upregulated in cluster 3

Gene Symbol	Gene Name	**Entrez Gene No**.	2 hr	6 hr	12 hr	24 hr	48 hr	72 hr
Nr4a2	nuclear receptor subfamily 4, group A, member 2	54278	1.60	**4.29**	1.30	**2.97**	**6.14**	**7.39**
Fosl1	fos-like antigen 1	25445	1.69	**2.09**	**2.15**	**2.34**	**3.02**	**6.46**
Itgav	integrin alpha V	296456	1.39	1.56	**2.78**	**2.38**	**5.32**	**5.22**
Twist2	twist homolog 2 (Drosophila)	59327	1.42	1.50	1.37	1.58	**3.08**	**4.84**
Rarb	retinoic acid receptor, beta	24706	0.89	0.83	1.42	1.80	**3.90**	**4.24**
Dusp1	dual specificity phosphatase 1	114856	0.96	1.70	1.89	1.47	**2.59**	**4.16**
Hmox1	heme oxygenase (decycling) 1	24451	1.63	**2.86**	**3.07**	**2.62**	**2.25**	**3.71**
Angptl4	angiopoietin-like 4	362850	1.23	1.28	1.33	1.98	**2.73**	**3.60**
Acin1	apoptotic chromatin condensation inducer 1	305884	0.92	0.67	1.31	**2.14**	**2.68**	**3.41**
Pdgfrb	Platelet derived growth factor receptor, beta polypeptide	24629	0.84	0.77	1.71	1.92	**2.99**	**3.26**
Gclc	glutamate-cysteine ligase, catalytic subunit	25283	1.25	1.99	1.58	1.80	**2.02**	**3.01**
Ihpk2	inositol hexaphosphate kinase 2	59268	1.07	1.17	1.26	1.64	**2.39**	**2.88**
Dapk1	death associated protein kinase 1	306722	0.85	0.95	1.55	1.38	**2.70**	**2.82**
Amigo2	adhesion molecule with Ig like domain 2	300186	0.87	0.93	1.00	1.33	1.49	**2.81**
Mmp2	matrix metallopeptidase 2	81686	1.05	1.12	1.32	1.48	1.92	**2.76**
Hipk2	Homeodomain interacting protein kinase 2	362342	1.01	1.63	2.14	1.86	**2.94**	**2.69**
Birc3	baculoviral IAP repeat-containing 3	78971	1.52	1.63	1.22	1.48	**2.27**	**2.68**
Myd116	myeloid differentiation primary response gene 116	171071	1.09	1.90	2.17	1.58	**2.95**	**2.67**
Il1rn	interleukin 1 receptor antagonist	60582	1.73	**2.51**	**3.06**	1.77	**3.57**	**2.49**
Hipk1	homeodomain interacting protein kinase 1	365895	1.12	1.05	1.30	1.37	**2.44**	**2.48**
LOC687118	similar to death effector domain-containing DNA binding protein 2	687118	1.58	1.58	**2.14**	**2.00**	**3.13**	**2.46**
Ripk2	receptor (TNFRSF)-interacting serine-threonine kinase 2	362491	1.04	1.33	1.48	1.69	**2.55**	**2.38**
Dapk1	death associated protein kinase 1	306722	0.97	1.00	1.16	1.35	**2.16**	**2.35**
Timp3	Tissue inhibitor of metalloproteinase 3	25358	1.16	1.08	1.28	1.01	1.78	**2.35**
Cln3	ceroid lipofuscinosis, neuronal 3, juvenile (Batten, Spielmeyer-Vogt disease)	293485	1.17	1.56	**2.08**	1.50	**2.44**	**2.32**
Ercc5	excision repair cross-complementing rodent repair deficiency, complementation group 5	301382	1.05	1.29	1.30	**2.06**	**2.13**	**2.28**
Pik3ca	phosphatidylinositol 3-kinase, catalytic, alpha polypeptide	170911	1.08	1.05	1.45	1.63	**2.07**	**2.20**
Raf1	v-raf-leukemia viral oncogene 1	24703	1.13	1.66	1.71	1.73	**2.01**	**2.17**
Ednrb	endothelin receptor type B	50672	1.32	1.09	1.46	1.94	1.31	**2.15**
Cd24	CD24 molecule	25145	0.78	0.61	0.70	0.71	1.39	**2.13**
Hipk2	homeodomain interacting protein kinase 2	362342	0.93	0.99	1.47	1.35	**2.33**	**2.09**
Timp3	Tissue inhibitor of metalloproteinase 3	25358	1.10	0.85	1.13	0.98	1.36	**2.08**
Vegfa	vascular endothelial growth factor A	83785	0.89	1.20	0.94	1.09	**2.02**	**2.07**
Gal	galanin	29141	1.32	1.22	1.85	0.77	1.84	**2.03**
Furin	furin (paired basic amino acid cleaving enzyme)	54281	1.17	1.20	1.36	1.18	1.89	**2.03**
Ep300	E1A binding protein p300	170915	1.33	1.09	1.23	1.29	**2.28**	**2.01**
Sh3kbp1	SH3-domain kinase binding protein 1	84357	1.03	1.58	1.78	1.47	**2.52**	1.85
Psen2	presenilin 2	81751	1.37	1.52	**2.14**	1.91	**2.09**	1.81
Ptk2b	PTK2 protein tyrosine kinase 2 beta	50646	0.93	1.29	1.95	1.69	**2.17**	1.81
LOC687813	similar to Tnf receptor-associated factor 1	687813	1.87	**2.36**	**2.35**	1.68	**2.71**	1.75
Ncf1	neutrophil cytosolic factor 1	114553	1.83	**2.78**	**2.72**	**2.44**	**2.59**	1.75
Apbb2	amyloid beta (A4) precursor protein-binding, family B, member 2	305338	1.09	1.12	1.48	1.28	**2.15**	1.74
Cln8	ceroid-lipofuscinosis, neuronal 8	306619	1.52	**2.11**	**2.21**	1.77	1.94	1.73
Sh3kbp1	SH3-domain kinase binding protein 1	84357	1.15	1.41	1.47	1.58	**2.41**	1.72

**Table 2:** List of "response to stimulus" genes selectively upregulated in cluster 1

Gene Symbol	Gene Name	**Entrez Gene No**.	2 hr	6 hr	12 hr	24 hr	48 hr	72 hr
Serpina3n	serine (or cysteine) peptidase inhibitor, clade A, member 3N	24795	1.53	**2.52**	**3.13**	**5.38**	**20.18**	**49.34**
Ereg	epiregulin	59325	1.99	**3.65**	**2.75**	**3.06**	**9.51**	**37.80**
Ptgs2	prostaglandin-endoperoxide synthase 2	29527	1.55	**3.81**	**2.35**	**3.48**	**12.52**	**21.58**
Ccl20	chemokine (C-C motif) ligand 20	29538	1.20	**2.27**	**2.07**	1.83	**8.52**	**20.38**
Cxcl3	chemokine (C-X-C motif) ligand 3	171551	1.62	**2.54**	**3.06**	**2.67**	**7.10**	**18.43**
Hspa4l	heat shock protein 4 like	294993	0.80	0.62	**2.19**	1.19	**4.98**	**7.69**
Grem1	gremlin 1	50566	1.11	1.45	1.12	**2.19**	**2.23**	**7.69**
Nupr1	nuclear protein 1	113900	0.93	0.81	1.69	1.34	**4.49**	**6.11**
Cryab	crystallin, alpha B	25420	0.83	0.88	1.35	1.06	**3.03**	**5.96**
Gls	glutaminase	24398	1.00	1.15	1.89	1.73	**3.80**	**5.44**
Cyp4b1	cytochrome P450, family 4, subfamily b, polypeptide 1	24307	0.72	1.75	1.26	0.66	1.42	**5.18**
Sncg	synuclein, gamma	64347	0.95	0.77	0.92	1.12	**2.72**	**4.71**
Vnn1	vanin 1	29142	1.10	1.37	1.43	1.31	1.64	**4.71**
Gls	glutaminase	24398	0.88	0.94	0.79	0.92	1.66	**3.89**
Cxcl10	chemokine (C-X-C motif) ligand 10	245920	1.23	1.01	0.84	1.08	1.65	**3.41**
Inhbb	Inhibin beta-B	25196	0.97	1.01	1.14	1.32	0.97	**3.28**
Slc12a2	solute carrier family 12 (sodium/potassium/chloride transporters), member 2	83629	0.88	0.88	0.97	0.88	1.58	**3.07**

### Identification of upregulated genes using Kyoto Encyclopedia of Genes and Genomes (KEGG) analysis

Based on the results of the clustering analysis, we tested the genes by all KEGG terms in clusters 1, 2 and 3 including those genes which were upregulated in a time-dependent manner under cyclic tensile stress. These pathways with p values < 0.05 are listed in Table 3. Four pathways were significantly included in cluster 1, 3 pathways in cluster 2, and 24 pathways in cluster 3.

**Table 3:** High frequency pathways* identified by KEGG/pathway analysis in clusters 1, 2, and 3

rank	pathway (total gene count)	p-value	count
cluster 1			
1	Glutamate metabolism (26)	0.0021	2
2	D-Glutamine and D-glutamate metabolism (3)	0.0081	1
3	Taurine and hypotaurine metabolism (10)	0.0266	1
4	Pantothenate and CoA biosynthesis (11)	0.0293	1
cluster 2			
1	T cell receptor signaling pathway (95)	0.0156	2
2	Glycosphingolipid biosynthesis - ganglio series (15)	0.0307	1
3	Cell adhesion molecules (CAMs) (151)	0.0371	2
cluster 3			
1	Focal adhesion (187)	0.0001	17
2	Bladder cancer (36)	0.0001	7
3	TGF-beta signaling pathway (81)	0.0003	10
4	Pathways in cancer (313)	0.0005	22
5	Prostate cancer (92)	0.0008	10
6	Small cell lung cancer (92)	0.0008	10
7	ECM-receptor interaction (74)	0.0028	8
8	Glioma (64)	0.0047	7
9	Melanoma (69)	0.0072	7
10	Renal cell carcinoma (69)	0.0072	7
11	PPAR signaling pathway (70)	0.0078	7
12	mTOR signaling pathway (54)	0.0081	6
13	Gap junction (90)	0.0091	8
14	Glycerophospholipid metabolism (46)	0.0168	5
15	MAPK signaling pathway (255)	0.0200	15
16	Non-small cell lung cancer (52)	0.0273	5
17	Pancreatic cancer (71)	0.0286	6
18	GnRH signaling pathway (91)	0.0296	7
19	Adherens junction (73)	0.0323	6
20	Pyruvate metabolism (38)	0.0351	4
21	Regulation of actin cytoskeleton (204)	0.0357	12
22	Leukocyte transendothelial migration (116)	0.0366	8
23	Chronic myeloid leukemia (79)	0.0449	6
24	Glutathione metabolism (42)	0.0482	4

*, Listed pathways are all statistically significant (P < 0.05). "total gene count" means the number of genes in each pathway, which have already been registered in KEGG system, and "count" means the number of genes, which were expressed significantly in each pathway in this study.

### Gene-specific real-time reverse transcription polymerase chain reaction (RT-PCR) in MAPK signaling pathway

**Figure 4:**
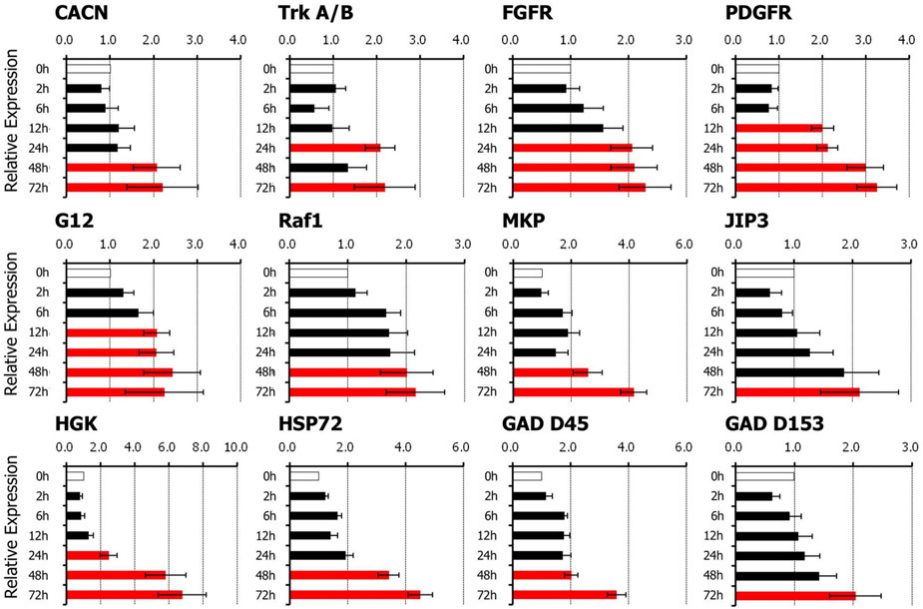
**The effects of cyclic tensile stress on gene expression levels analyzed by real time RT-PCR**. Application of cyclic tensile stress resulted in significant increases in mRNA expression levels of platelet derived growth factor receptor (PDGFR), guanine nucleotide binding protein gamma 12 (G12) at 12 hours, neurotrophic tyrosine kinase receptor type 2 (trkA/B), fibroblast growth factor receptor (FGFR), mitogen-activated protein kinase kinase kinase kinase 4 (HGK) at 24 hours, and calcium channel voltage-dependent L type alpha 1F subunit (CACN), v-raf-leukemia viral oncogene 1 (Raf1), dual specificity phosphatase 1 (MKP), mitogen-activated protein kinase 8 interacting protein 3 (JIP3), heat shock protein 72 (HSP72), DNA-damage-inducible alpha (GAD D45), DNA-damage inducible transcript 3 (GAD D153) at 48-72 hours. Red bars indicate significant differences (P < 0.05) in upregulation at least 2-fold in comparison to controls. Data are mean ± SEM of 3 experiments.

In further examination of KEGG analysis of cluster 3, which included many of the upregulated genes, we found that the MAPK signaling pathway contained 12 candidate genes among the 15 genes that were significantly upregulated. These genes were calcium channel voltage-dependent L type alpha 1F subunit (Cacnalf or CACN), neurotrophic tyrosine kinase receptor type 2 (Ntrk2 or trkA/B), fibroblast growth factor receptor 2 (Fgfr2 or FGFR), platelet-derived growth factor receptor, beta polypeptide (Pdgfrb or PDGFR), v-raf-leukemia viral oncogene 1 (Raf1 or Raf), guanine nucleotide binding protein (G protein) gamma 12 (Gng12 or G12), dual specificity phosphatase 1 (Dusp1 or MKP), mitogen-activated protein kinase kinase kinase kinase 4 (Map4k4 or HGK), mitogen-activated protein kinase 8 interacting protein 3 (Mapk8ip3 or JIP3), heat shock protein 72 (Hspa 72 or HSP72), growth arrest and DNA-damage-inducible alpha (Gadd45a or GAD D45), and DNA-damage inducible transcript 3 (Ddit3 or GAD D153). To confirm the expression of genes identified using the microarrays, these 12 genes were identified to test differential expression using real time RT-PCR analysis. The mRNA expression levels of PDGFR, G12 was significantly increased from the mid period of application of cyclic tensile stress (12-hour stress duration), while CACN, trkA/B, FGFR, Raf1, MKP, HGK, JIP3, HSP72, GAD D45, and GAD D153 mRNA expression levels increased during the late phase of cyclic tensile stress (24-72 hours duration). In each case these significant upregulations in mRNA expression levels was by at least 2-fold in comparison with the control levels at time 0 (Figure 4).

## Discussion

In vitro systems offer several advantages over in vivo systems in the analysis of cellular responses to their mechanical environment, including the precise specification of loading parameters (strain levels and strain rate), control of the extracellular environment (temperature, ion concentration, partial pressure of gases), the relative ease and repeated access of the cells, and simplified administration of pharmacological compounds. By precisely controlling the loading conditions, the quantitative relationship between the severity of mechanical injury and response to the injury can be evaluated [24]. Previous mechanical stimulation studies of neuronal cells using immortalized cell lines reported that the physiological strain conditions of neuronal cells are not as high as osteoblasts or skeletal muscle cells [25]. Rat motoneurons stretched at 11% strain showed ischemic changes but no mechanical damage [26], whereas at a 6% strain level there were no ischemic changes or mechanical damage [27]. PC12 cells were subjected to cyclic tensile strain levels ranging from 4 to 16% at strain frequencies of 1-2 Hz as physiological mechanical conditions [25]. Based on these reports and considering primary cultured spinal cord cells [23], we selected the range of cyclic tensile stresses most appropriate to our culture system in the present study, and the cells were observed morphologically following application of various tensile strains levels (5%, 10%, and 15%) and strain frequencies (0.5 Hz and 1 Hz) using the Flexercell system. Interestingly, we found a significant decrease in the cell survival rate (%) when cultures were subjected to either a cyclic tensile stress of 5% at 1 Hz or 15% at 0.5 Hz, compared with a cyclic tensile stress at 10% at 0.5 Hz, which was taken as a control level. Our results suggest that both the level of strain applied and frequency of its application influence cell viability. Furthermore the results demonstrate that a higher strain level at a lower strain rate can have a similar effect as a lower strain level at a higher strain rate in neuron-rich spinal cord cells.

To understand the molecular mechanism of neuronal responses to cyclic tensile stress, DNA microarray was employed to identify the specific gene expression patterns in cultured spinal cord cells, and the hierachical clustering algorithm was used in the analysis. Consequently, we found that 44 genes related to "apoptosis" among cluster 3 included the majority of progressively upregulated genes by enrichment analysis using biological process in the GO system. Furthermore, 17 genes related to "response to stimulus" were also identified among those of cluster 1, including most upregulated genes in the late phase after application of cyclic tensile stress. On the other hand, the KEGG/pathway analysis identified different pathways in the upregulated genes in a time-dependent manner in the clusters 1, 2, and 3 which had been identified via the GO system.

A number of investigators have attempted to characterize or determine changes in specific genes and signaling pathways of direct mechanical stress or load using cells from diverse tissues and in vitro models of mechanical trauma. These systems are multifold and can be separated into detection, response, and modulation pathways [28]. A load stimulus is detected by a mechano-electrochemical sensory system, including mechanically sensitive ion channels (stretch-activated channels) linked to the cytoskeleton [29]. The principle detection system is the matrix-integrin-mechanosensory protein complex-cytoskeleton machinery [30,31]. This system is linked to the kinase cascade (tyrosine or nontyrosine kinase cascade or the JAK/STAT kinase cascade) system, which provides amplification, diversity, selectivity, and modulation capabilities and contains focal adhesion kinase (FAK) [32]. The Src homology protein complex (SHC) and growth factor receptor binding adaptor protein linking receptor (GRB2) link to the Ras-raf signaling pathway, and these are regulated through FAK. Ras and GTPase activation regulates the activation of Raf, MAPK and extracellular regulated kinase (ERK) [33,34]. Activated ERKs enter the nucleus and upregulate the expression of various transcription factors (jun, fos, myc, erg-1) and activate nuclear binding proteins such as NF-κB [35]. Specific regulation may occur at load response elements in promoters of certain genes such as platelet-derived growth factor (PDGF) [36]. The mechanically sensitive ion channel could also be a gap junction channel, which propagates a Ca^2+ ^wave from cell to cell after a mechanical signal is detected through a cell adhesion molecule (CAM).

It is clear that systems involved in evoking the response of neuronal cells to applied mechanical load are highly complex, which requires an expansive approach to their elucidation. Our results in primary neuronal-rich culture have analyzed gene expression profiles using the Affymatrix system and thereby have identified 3,412 genes with altered expression as a result of applied cyclic tensile stress, many of which are consistent with previous studies. These data will be useful for future examination of how the effects of mechanical insult on the spinal cord, in particular, are regulated. Signalling pathways that may be involved in this process can be modulated by molecules such as G proteins, kinase phosphorylation and activation, and kinase inhibitors or phosphatases [34]. Indeed, the MAPK kinase pathway was identified by KEGG analysis of the alterations in gene expression seen in the mechanically loaded spinal cord cells. MAPKs are a family of related serine/threonine protein kinases that transduce several signals responsible for cell proliferation or cellular stress, and are also intracellular signaling systems that induce optimal stress responses. In general, MAPK is activated by phosphorylation of tyrosine and threonine residues by MAPKK, which is activated through phosphorylation by MAPKKK. In KEGG analysis, the MAPK signaling pathway can be classified into three main groups [37]: the classical MAP pathway, the c-Jun N-terminal kinase or stress-activated protein kinase (JNK), the p38 MAPK signaling pathway, and the ERK pathway. In the present study, the mRNA expression levels of PDGFR and G12 significantly increased during the mid period of cyclic tensile stress application, while CACN, Raf1, MKP, JIP3, HSP72, GAD D45, and GAD D153 mRNA levels increased during the late phase of the cyclic tensile stress application. These genes, which were further identified by real time RT-PCR analysis, may play an important role in the response of spinal cord cells to neuronal injury. Previous *in vivo *studies of spinal cord injuries using microarray have also demonstrated the participation of similar genes in acute phage after injury [9,13]. Gene expression of trkA/B, FGFR, PDGFR, and Raf1 could facilitate neuronal survival, while gene expression of G12, HSP72, GAD D45, GAD D153 could be involved in DNA damage. Further studies that target specific gene expression pathways will help determine their precise role in neuronal responses to mechanical load.

Our study is perhaps the first to attempt investigating the expression of genes related to apoptotic cell death during the application of cyclic tensile stress to neuronal rich spinal cord cells; however, it has several limitations. These include (i) imperfect validity of microarray results, with a CV of 5-15% for quantitative signals [38]; (ii) the neuronal culture was not 100% pure (the rate of NeuN-positive cells was 71%), and embryonic cells were used, not adults cells; and (iii) the lack of immunohistological or in situ hybridization data to identify the exact types of cells in which alterations in gene expression occurred. Nevertheless, we believe that the present study might be the first to comprehensively profile changes in gene expression involved in neuronal responses to cyclic tensile stress in cultured spinal cord cells using DNA microarray. This is a considerable improvement in examining the specific response of neuronal cells to mechanical load. Considered together with our previous findings [23], we can conclude that certain apoptosis-specific genes are activated in neuronal cell rich cultures during the application of cyclic tensile stress. The clinical relevance of tensile stress may specifically include the tethering effect with the developmental ascensus medullaris [23], cervical myelopathy in association with kyphotic deformity [4], and complicated spinal cord distraction injury. However, it is perhaps intuitive to consider that abnormal tensile stresses are involved in many mechanical insults of the spinal cord. Thus, we believe that our study provides new insights into the pathophysiology of spinal cord damage in various disease entities. Furthermore, the current study may help understand the response of neuronal cells to cyclic tensile stress and therapeutic issues related to the mechanically damaged spinal cord.

## Conclusions

We have investigated the effects of cyclic tensile stresses on cultured spinal cord cells and demonstrate that cell death was induced depending on the level and duration of strain applied. Furthermore, we have performed a comprehensive analysis of alterations in gene expression profiles that occur following this mechanical stress, and identified in particular an upregulation of members of the MAPK pathway. Knowledge of the specific response of neuronal cells to mechanical insult could be a potentially useful tool for molecular-based therapy for spinal cord injury.

## Methods

### Cell isolation and culture

Primary cultures were established using the method described previously by our group [23]. In brief, the spinal cords of Sprague-Dawley rat embryos were dissected out at post-coital day 15 and stripped of the dorsal root ganglia and meninges. Dissected tissues were rinsed with cold Ca^2+^- and Mg^2+^-free Hanks balanced salt solution (HBSS) supplemented with 4 g/L glucose, and incubated at 37°C for 20 minutes with 0.03% (w/v) trypsin solution in HBSS under mild shaking. They were transferred into HBSS containing 0.1% (w/v) soyabean trypsin inhibitor (Sigma, St. Louis, MO) and 0.2% (w/v) bovine serum albumin (BSA), and triturated very mildly. The cell suspension was filtered through nylon mesh (70 μm, Cell Strainer; Becton Dickinson, Bedford, MA). The culture medium consisted of 75 mL Leibovitz's L-15 medium supplemented with glucose (4 g/L), 1.0 mL N2 supplement, 15 mL 0.15 M sodium bicarbonate, 10 mL heat-inactivated horse serum, 1 mL of 100 mM L-cysteine and 1 mL penicillin G 10^4 ^U/mL and neutralized with CO2. After centrifugation at 400 × g for 15 minutes at 4°C, the precipitated cells were gently re-suspended in a fresh culture medium and plated at a density of 4.0 × 10^5 ^cells/well onto a 6-well culture plate with a flexible-polystyrene bottom coated with type IV collagen (BioFlex^® ^Baseplate, Flexercell International).

The experiment was carried out in the Orthopaedic Spinal Cord Laboratory of our University Medical Faculty. The experimental protocol strictly followed the Ethics Review Committee Guidelines for Animal Experimentation of our University.

### Application of cyclic tensile stress to the cultured spinal cord cells

The cell stretching device used in this study was the Flexercell FX3000^® ^(Flexercell International). The device consists of a computer-controlled vacuum unit, a culture plate with a flexible-polystyrene well-bottom coated with type IV collagen (BioFlex^® ^Baseplate), and another culture plate with a non-deformable culture well bottom constructed of the same material as control. The culture plates consisted of 6-well (35 mm diameter) flexible-bottomed culture plate with a hydrophilic surface. The application of a vacuum provides a hemispherically downward deforming force onto the flexible bottom, resulting in a non-homogenous strain profile with a maximum at the periphery and a geometric decline toward zero at the center of the culture well bottom. For these experiments, the cultured spinal cord cells were subjected to various conditions. The flexible-bottomed culture plates including the control plates were then placed on the vacuum baseplate in the incubator (37°C in 5% CO2). Three days after cell seeding, the cells were subjected to cyclic tensile stress for up to 72 hours. Previously we have documented [23] that ~71% of cells isolated from the spinal cord using this methodology are positive for the neuronal marker NeuN (Chemicon International, Temecula, CA). Repeated examinations by phase contrast microscopy showed that the cells remained attached to the substratum during elongation of the flexible-polystyrene well plates as tensile stresses were applied. Panjabi and White [16] demonstrated that the elastic properties of the cervical spinal cord are dramatically altered when it is subjected to approximately 10% elongation. In our previous *in vivo *study [17], the amplitude of epidurally-recorded spinal cord evoked potentials began to diminish, especially the second component (N2 spike), when the longitudinal extension of the cord shortened by 10-17%. Thus, in the present study, we set the tensile stress levels at a maximum 15% elongation and set the strain rate to estimate an appropriate frequency of spine movement in everyday life. Therefore, the cells were observed morphologically following application of various tensile stresses resulting in strains of 5%, 10%, and 15% applied at frequencies of 0.5 Hz and 1 Hz. Analyses of cell survival, DNA microarrays and real-time RT-PCR were conducted at 0, 2, 6, 12, 24, 48 and 72 hours after the application of the cyclic tensile load. DNA microarray analysis and real time RT-PCR were performed to compare the levels of gene expression at time 0 with levels of gene expression thereafter after the cyclic tensile loading at o.5 Hz, resulting in 10% strain.

### Quantification of cell survival under cyclic tensile stress

Cell survival was investigated by scoring the number of living cells after tensile stress application using the Live/Dead Assay (Live/Dead Assay, Molecular Probes, Eugene, OR), according to the manufacturers' instruction. This assay if based on the differential staining of cells with calcein-acetoxymethyl ester (calcein AM: 3.00 μM) to identify living cells and ethidium homodimer-1 to identify dead cells [39,40]. Calcein-AM is a membrane-permeable dye that is cleaved by intracellular esterase to produce an impermeant green-wavelength fluorophore in living cells. Ethidium homodimer-1 cannot penetrate live cells, but it can enter dead cells which have a porous membrane and hence bind to DNA to produce red fluorescence. The culture medium was removed and the cells were then washed twice with PBS, and stained for 75 minutes at 32°C. The numbers of attached living cells (green) in at least 6 high-power fields (each containing at least 100 cells) were counted using fluoromicroscopy (IX70, Olympus, Tokyo) and a color image analyzer (MacSCOPE, Mitani, Fukui, Japan) in more than three wells for each time point. There was no evidence of spinal cord cell proliferation during the 3 day period prior to treatment with cyclic tensile stress, i.e. cell counts were almost uniform and at a density between 3.3 × 10^5 ^and 4.8 × 10^5 ^cells/well after dissemination on Bioflex^® ^Baseplate in the absence of mechanical stimuli. The cell survival rate (%) at each time point for cultures which were subjected to 10% strain at 0.5 Hz frequency was calculated relative to the cell number at 0-hour. These cultures (subjected to 10% tensile strain at 0.5 Hz frequency) were then set as a standard, to which the cell viability of other levels of strain and frequency were compared.

All values were expressed as mean ± standard error of the mean (SEM). Differences between values of the loaded and control cultures were tested at each point by one-way ANOVA and Tukey posthoc test using the SPSS software version 11.0 (SPSS, Chicago, IL). P values of less than 0.05 denoted the presence of a statistically significant difference.

### TEM examination

The presence of DNA fragmentation was examined via TEM examination. After application of tensile stresses, cultured spinal cord cells were washed twice with PBS and fixed with 2.5% glutaraldehyde and 2.5% paraformaldehyde, followed by late fixation in 1% osmium tetroxide for 2 hours. Fixed specimens were dehydrated in a graded series of alcohol, embedded in epoxy resin and polymerized at 60°C for 2 days. Ultrathin sections were cut by ultramicrotome, stained with uranyl acetate and lead citrate, and examined with a Hitachi H-7650 TEM (Hitachi, Tokyo).

### RNA preparation and DNA microarray hybridization

The cultured cells on each well at 0, 2, 6, 12, 24, 48 and 72 hours were disrupted in a lysis buffer containing β-mercaptoethanol and the total RNA from five animals was pooled at each time point, and further purified using RNeasy^® ^Mini Kit (Qiagen, Valencia, CA) and treated with DNase I (TaKaRa Bio, Ohtsu, Japan). The quality of RNA was initially assessed by electrophoresis on a 1.5% agarose gel, and further determined by using the RNA 6000 Nano LabChip Kit^® ^and Agilent Bioanalyzer 2100 (Agilent, Palo Alto, CA). cDNAs were synthesized by GeneChip T7-Oligo (dT) Promoter Primer Kit^® ^(Affymetrix, Santa Clara, CA) and TaKaRa cDNA Synthesis Kit^® ^(TaKarRa Bio) from 3 μg total RNA. Biotinylated cRNA were synthesized using the IVT Labeling Kit (Affymetrix). Following fragmentation, 20 μg of cRNA were hybridized for 16 hours at 45°C on the GeneChip^® ^Rat Genome 230 2.0 Array. GeneChips were washed and stained in the Affymetrix Fluidics Station 450, and scanned using GeneChip Scanner 3000 7G.

### Data analysis of GeneChip^® ^expression array

Microarray data was initially processed using GeneChip Operating Software (GCOS, Affymetrix). Signal intensity was calculated by Microarray Suite version 5.0 (MAS5.0) with Affymetrix default setting and global scaling as the normalization method. The trimmed mean target intensity of each array was arbitrarily set to 500. The ratio and difference of intensity between two corresponding genes on each array was calculated. Significance of the difference in levels of gene expression was set at a ratio and difference of intensity of ≧2 and ≧200, respectively. Spots that could not be interpreted were excluded, resulting in a list of 3,412 genes available for subsequent analysis. After filtering, the hierarchical clustering was applied to the axis using the complete linkage method, as implemented in the program Spotfire 8.1.1. The distance matrices used were uncentered, with correlation calculated on the basis of expression signals for clustered genes.

### GO and KEGG analysis

We used the GO system and KEGG system for categorizing identified genes, and identified specific genes and pathways in each category by enrichment analysis. The Gene Ontology Consortium (http://www.geneontology.org/doc/GO.doc.html) maintains a controlled vocabulary database of functional descriptions for genes. These are divided into three families: biological process, cellular component, and molecular function. We searched for GO terms associated with Gene ID for GeneChip^® ^Rat Genome 230 2.0 Array and were able to associate 3,540 of them with GO terms (GO numbers). In our study, we used the total list of GO terms within the biological process categories. The number of times a GO term (or group of terms) appeared at each sub-level of the GO tree was counted, and hierarchical sums were calculated (of the number of occurrences at or below each sub-branch). The KEGG system (http://www.genome.jp/kegg/) provides a reference knowledge base for linking genomes to life through the process of pathway mapping. We calculated a probability to determine whether any GO terms or pathways annotate a specified list of genes at a frequency greater than that would be expected by chance. The probability was determined using the following hypergeometric distribution formula:

where N is the total number of genes, M is the number of genes that annotated for a given term or pathway, n is the size of the list of genes of interest and k is the number of genes within the list that annotated for a given term or pathway.

Microarray including GeneChip^® ^loads some different prove sequences in order to identify one gene. Strictly speaking, microarray data based on signal intensity represents the loading common probe set domain designed by Affymetrix. Therefore, it is possible that the number of gene symbols is different from that of the identified gene by the probe set.

### Real-time RT-PCR analysis

Reverse transcription was performed using 500 ng of total RNA, AMV reverse transcriptase XL (TaKaRa Bio) and random primer. Real-time PCR was performed on the PRISM 7000 (ABI) system using 1 μl of the synthesized cDNA and SYBR Green PCR master mix (Applied Biosystems, Foster, CA). Table 4 lists the primer sequences used in the present study. The target genes were amplified and analyzed in triplicate using ABI Prism 7000 SDS Software (Applied Biosystems). The expression levels of target genes were normalized to that of glyceraldehyde-3-phosphate dehydrogenase (GAPDH) at each time interval, and the relative expression levels of target genes were calculated relative to that at 0 hour.

**Table 4:** Sequences of primers used for real-time PCR

Target Protein	Forward Primer	Reverse Primer	PCR Product Size (bp)	**Sequence Accession No**.
CACN	5'-TCACCATTGCCTCCGAACACTA-3'	5'-CAGGAGCATTTCTGCCGTGA-3'	104	NM012517
TrkA/B	5'-GCCACACAATGTTGCCCATC-3'	5'-AAGGACTCTGCCCTGGGTGA-3'	185	NM012731
FGFR	5'-TTGCCGAATGAAGACCACGA-3'	5'-GGAGTTCATGGACGAGCTGGA-3'	130	NM001109892
PDGFR	5'-GAATGACCACGGCGATGAGA-3'	5'-GGATAAGCCTCAAACACCACCTG-3'	141	NM031525
G12	5'-CAAGATGCTGGTGGCTGTCAA-3'	5'-AGCAGCTCTGCCTCACGATG-3'	81	NM021589
Raf1	5'- AACAGTGAAGTCGCGCTGGA-3'	5'- CAGCACAATGCCATAGGAGTAGACA-3'	145	NM012639
MKP	5'-ACAACCACAAGGCAGACATTAGCTC-3'	5'- CAGATGGTGGCTGACCTGGA-3'	127	NM053769
JIP3	5'-AGCCTGCCTGCCAAGTACAAG-3'	5'-TCAGGTTGACACCAGCAGCAC-3'	148	NM001100673
HGK	5'-TTCACCATGTCATTCACCGAGA-3'	5'-CCAGCTGAGCGCTTACACCA-3'	99	NM001106904
HSP72	5'-GCTTTCACCTCAAGCCTTTGGA-3'	5'-CGGGCCTCATGCACACATAG-3'	117	NM212504
GAD D45	5'-AGGCAGCCAAGCTGCTCAA-3'	5'-ACGTCCCGGTCGTCATCTTC-3'	82	NM024127
GAD D153	5'-TGGAAGCCTGGTATGAGGATCTG-3'	5'-GAGGTGCTTGTGACCTCTGCTG-3'	175	NM024134
GAPDH	5'-GGCACAGTCAAGGCTGAGAATG-3'	5'-ATGGTGGTGAAGACGCCAGTA-3'	143	NM017008

## Competing interests

The authors declare that they have no competing interests.

## Authors' contributions

KU and HB designed the study and drafted the manuscript. KU carried out all the experiments and performed the statistical analysis. HN and TH participated in the microarray analysis. TY, KC and SK participated in the experiments of cell morphology and viability. WEJ conceived the study, participated in its design and coordination and SR helped to draft the manuscript. All authors read and approved the final manuscript.
